# Stomatitis and VEGFR-Tyrosine Kinase Inhibitors (VR-TKIs): A Review of Current Literature in 4369 Patients

**DOI:** 10.1155/2018/5035217

**Published:** 2018-05-24

**Authors:** Claudia Arena, Giuseppe Troiano, Alfredo De Lillo, Nunzio F. Testa, Lorenzo Lo Muzio

**Affiliations:** Department of Clinical and Experimental Medicine, University of Foggia, Foggia, Italy

## Abstract

**Background:**

Multitargeted tyrosine kinase inhibitors (TKIs) represent a new class of target-specific antineoplastic agents. These agents show some specific adverse events such as fatigue/asthenia, anorexia/loss of appetite, dysgeusia, diarrhea/abdominal pain, hypothyroidism, hypertension, myelosuppression, and stomatitis.

**Materials and Methods:**

A systematic search was performed on PubMed online database using a combination of MESH terms and free text words, “sunitinib” OR “sorafenib” OR “axitinib” OR “cabozantinib” OR “pazopanib” OR “regorafenib” OR “nintedanib” OR “vatalanib” combined through the use of Boolean operator AND with the key words “stomatitis” OR “mucositis,” (i) on human subjects, (ii) written in the English language, and (iii) reporting about the incidence of stomatitis or oral mucositis.

**Results:**

The incidence of stomatitis of any grade was 35.2% for sunitinib, 20.52% for sorafenib, 20.63% for axitinib, and 34.21% for cabozantinib. All the agents showed high rates of low-grade stomatitis (G1-G2), while the onset of severe stomatitis (G3-G4) was very low.

**Conclusions:**

Analysis of the reports with patients treated with sunitinib, sorafenib, axitinib, and cabozantinib showed a clear prevalence of stomatitis grade 1 or grade 2. These data differ from those of patients treated with conventional chemotherapy in which mucositis is predominantly of grade 3 or grade 4.

## 1. Introduction

Traditional treatment of malignancies with chemotherapeutic agents often causes the damage of normal healthy cells [1]. Toxicities of the oral cavity, such as mucositis and stomatitis, are some of the most significant and unavoidable side effects associated with cancer treatment [2]. Oral toxicities have a huge impact on the patient with cancer and are a common cause of dose delays and interruptions of cancer therapy [3]. The terms “oral mucositis” and “stomatitis” are often used interchangeably to indicate oral complications of anticancer therapy, but they do not refer to the same process (Parkhill, 2013). Oral mucositis is a Medical Subject Headings term that describes inflammation of oral mucosa due to chemotherapeutic agents or ionizing radiation. Stomatitis is a less specific term used to describe any inflammatory condition of oral tissue. For such reason in the last decades, newer targeted agents have been developed, aiming to decrease the rates of side effects on healthy cells.

Multitargeted tyrosine kinase inhibitors (TKIs) represent a novel class of target-specific antineoplastic agents. The mechanism of action of this class of drugs is based on the block of several key tyrosine kinase pathways in human cancers, including the vascular endothelial growth factor receptor (VEGFR), epidermal growth factor receptor (EGFR), human epidermal growth factor receptor 2 (HER2), and platelet-derived growth factor receptor (PDGFR) [4–6]. Molecules that inhibit VEGFR-Tyrosine Kinase Inhibitors (VR-TKIs) are an emerging class of highly effective targeted therapies due to their demonstrated efficacy in a variety of malignancies [5, 7–12]. FDA-approved VR-TKIs include sorafenib (renal cell carcinoma [RCC], hepatocellular carcinoma [HCC], and thyroid cancer), sunitinib (RCC, HCC, and gastrointestinal stromal tumor [GIST]), pazopanib (RCC and soft tissue sarcomas), cabozantinib (metastatic medullary thyroid cancer), and regorafenib (GIST and colorectal carcinoma [CRC]) [9, 10, 13–17].

Even this kind of targeted therapy based on VR-TKIs showed some class-specific adverse events that include fatigue/asthenia, anorexia/loss of appetite, hand-foot skin reaction, stomatitis, dysgeusia, diarrhea/abdominal pain, hypothyroidism, hypertension, and myelosuppression [63–66]. Literature reported that 25% of patients treated with multitargeted angiogenesis kinase inhibitors develop an oral adverse event within 2 months of therapy [67].

## 2. Materials and Methods

The following review was performed to answer to the following question: “which is the rate of incidence of oral stomatitis in patients treated with VEGF TKIs?”

A systematic search was performed on the PubMed online database using a combination of MESH terms and free text words, “sunitinib” (free text) OR “sorafenib” (free text) OR “axitinib” (free text) OR “cabozantinib” (free text) OR “pazopanib” (free text) OR “regorafenib” (free text) OR “nintedanib” (free text) OR “vatalanib” (free text) combined through the use of Boolean operator AND with the key words “stomatitis” (MESH) OR “mucositis” (MESH), (i) performed on human subjects, (ii) reporting about the use of an mTOR inhibitor, (iii) written in the English language, and (iv) reporting about the incidence of stomatitis or oral mucositis.

Case reports and studies on animal model were excluded from this study. No restrictions were applied to the year of publication.

For each study, the following records were extracted: name of the first author, year of publication, number of patients enrolled, type of disease treated, number of events recorded, and grade of the events reported. To simplify the process of data extraction, an ad hoc extraction sheet was used. In addition, data were independently extracted by two authors (Lorenzo Lo Muzio and Claudia Arena) and checked in a joint session.

## 3. Results

### 3.1. Bibliographic Research

**Figure 1:**
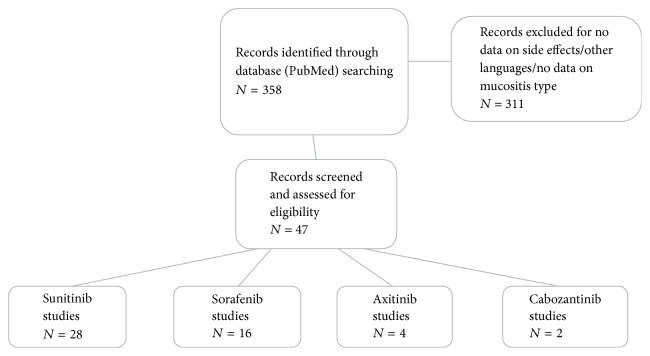
Flow chart showing the process of papers selection used in this review.

Titles and abstract of 358 potentially relevant studies were screened; of these, 311 studies were excluded because they did not meet the inclusion criteria (Figure 1). The full texts of 47 studies were read. Of the included studies, 28 referred to sunitinib use, 16 to sorafenib, 4 to axitinib, and 2 to cabozantinib. Of these, 5 referred to both sunitinib and sorafenib and 2 referred to both axitinib and sorafenib.

### 3.2. Analysis of Data

For sunitinib, 28 studies were analyzed (Table 1). A total of 2.596 patients were treated with sunitinib. The overall incidence of stomatitis of any grade with treatment was 35.2% (914 patients). Studies reported data about grade of stomatitis for 2068 patients and 739 cases were grade 1/2 (35.73%) and 90 were grade 3/4 (5.35%).

For sorafenib, 16 studies were analyzed (Table 2). A total of 1218 patients were treated with sorafenib. The overall incidence of stomatitis of any grade with treatment was 20.52% (250 patients). Studies reported data about grade of stomatitis for 830 patients and 174 cases were grade 1/2 (20.96%) and 19 were grade 3/4 (2.28%).

For axitinib, 4 studies were analyzed (Table 3). A total of 441 patients were treated with axitinib. The overall incidence of stomatitis of any grade with axitinib treatment was 20.63% (91 patients) and 79 cases were grade 1/2 (17.91%) and 12 were grade 3/4 (2.72%).

For cabozantinib, 2 studies were analyzed (Table 4). A total of 114 patients were treated with cabozantinib. The overall incidence of stomatitis of any grade with cabozantinib treatment was 34.21% (39 patients) and 34 cases were grade 1/2 (29.82%) and 5 were grade 3/4 (4.38%).

## 4. Discussion

Targeted therapy is a kind of chemotherapy that inhibits a molecular target which is abnormally expressed in malignancy. This method allows reaching a preferential localization of a drug in the region of disease, thus achieving an increase in local concentration. VEGFR TKI drugs work by inhibiting neoangiogenesis in the tumor.

The cloning of vascular endothelial growth factor in 1989 was a major step in understanding of tumor angiogenesis. Angiogenesis inhibitors are a class of drugs that include monoclonal antibodies and tyrosine kinase inhibitors.

In this review, we focused on oral side effects provoked by tyrosine kinase inhibitors. Small molecule inhibitors of VEGFR2 were first reported in 1996. This type of therapy is based on the fact that tumor cells can obtain the necessary oxygen and nutrients for survival by passive diffusion for tumor size <1-2 mm, but angiogenesis is necessary for tumor growth beyond the size of 100–300 cells [68]. The mRNAs for both VEGFR1 and VEGFR2 are reported to be upregulated in tumor-associated endothelial cells in comparison to the vasculature of the surrounding normal tissue. Moreover, recent studies highlighted that VEGF and VEGFR-1 and VEGFR-2 not only drive tumor angiogenesis but also directly stimulate tumor growth and the formation of metastases [69].

Overexpression of both VEGF and VEGFR is reported for many human solid cancers, including cancers of the gastrointestinal tract [70, 71], pancreas [72], breast [73, 74], stomach [75], cervix [76, 77], bladder [78, 79], kidney [78], prostate [80], ovaries [81, 82], endometrium [83], lung [84], brain [85, 86], and melanoma [87] and squamous cell carcinoma of the head and neck [88].

The main oral side effects reported in the studies include nonspecific stomatitis, dysgeusia, and xerostomia. These toxicities may occur alone or in combination.

Results of analysis of the literature showed that the incidence rate of overall stomatitis is higher in patients treated with sunitinib (40.08%) compared to sorafenib (22.55%), axitinib (20.63%), and cabozantinib (34.21%). Although it was not possible to carry out an accurate analysis of stomatitis by grade, it can be noted that most of the studies included in the review showed a high rate of minor stomatitis (G1-G2), while the onset of severe stomatitis (G3-G4) was lower. Indeed, in patients treated with sunitinib, the rate of incidence of low-grade stomatitis was 35.73%, while the rate of incidence of high-grade stomatitis was 5.35%; in patients treated with sorafenib, the rate of incidence of low-grade stomatitis was 18.67%, while the rate of incidence of high-grade stomatitis was 2.28%; in patients treated with axitinib, the rate of incidence of low-grade stomatitis was 17.91%, while the incidence of high-grade stomatitis was 2.72%; in patients treated with cabozantinib, the rate of incidence of low-grade stomatitis was 29.82%, while the rate of incidence of high-grade stomatitis was 4.38%. These results differ from those reported in literature about mucositis provoked by conventional chemotherapy in which mucositis is often a severe and dose-limiting toxicity.

The stomatitis caused by this kind of targeted therapy presents as a diffuse mucosal hypersensitivity/dysesthesia which can be associated with moderate erythema or inflammation of the oral mucosa. The symptoms appear in the first week of treatment and then gradually disappear [67, 89]. The literature reports that sunitinib and sorafenib may cause linear lingual ulcers of the nonkeratinized mucosa. Other typical oral side effects caused by treatment with VEGFR TKI are dysgeusia reported after treatment with cabozantinib and sunitinib and benign migratory glossitis which can be moderately painful and usually does not require any treatment modification or specific local treatment [90].

The changes in vascular permeability caused by the inhibition of VEGF can also induce mucocutaneous bleeding [91] and a delay in wound healing. Moreover, an oral screening for patients should be considered before undergoing therapy with antiangiogenic treatment. Treatment with tyrosine kinase inhibitors should end at least 1 week before oral surgery.

## 5. Conclusion

In conclusion, the targeted therapy has not kept the initial promises, as it determines several side effects, even if it is often lower than traditional chemotherapy. Regarding the oral cavity, the main side effect remains stomatitis, present in 20–30% of patients. The major advantage is that stomatitis is predominantly grade 1-2 in patients treated with targeted therapy while the effects of conventional chemotherapy are predominantly grades 3 and 4.

## Figures and Tables

**Table 1:** Report on all papers about sunitinib and stomatitis.

	Authors	Year	Neoplasia	Number of cases	Stomatitis number	Stomatitis grade 1	Stomatitis grade 2	Stomatitis grade 3	Stomatitis grade 4
(1)	Arakawa-Todo et al. [18]	2013	Metastatic renal cell carcinoma A: sunitinib 50 mg once daily was given in repeated 6-week cycles of 4 weeks followed by 2 weeks off	A: 15	TOT: 9 (60%)	A: 9 (60%)		A: 0	

(2)	Armstrong et al. [19]	2016	Metastatic nonclear renal cell carcinoma A: sunitinib 50 mg/day; 6-week cycles of 4 weeks with treatment followed by 2 weeks without treatment	A: 51	A: 14 (27.45%)	A: 14 (27%)		A: 0	

(3)	Bang et al. [20]	2011	Advanced gastric cancer A: sunitinib 50 mg/day for 4 weeks on treatment and 2 weeks off	A: 78	A: 28 (35.9%)	A: 27 (34.6%)		A: 1 (1.3%)	

(4)	Cardoso et al. [21]	2012	HER2-positive metastatic breast cancer A: sunitinib 37.5 mg (starting dose across sunitinib combination studies) once daily by oral capsule on schedule 2/1	A: 25	A: 12 (48%)	A: 9 (36%)		A: 3 (12%)	

(5)	Carrato et al. [22]	2013	Metastatic colorectal cancer randomized phase III trial A: sunitinib plus FOLFIRI (fluorouracil, leucovorin, and irinotecan)	A: 386	A: 35 (9%)	Not reported		A: 35 (9%)	

(6)	Dirican et al. [23]	2013	Metastatic renal cell carcinoma A: sunitinib: 50 mg per day was administered in repeated 6-week cycles of daily therapy for 4 weeks, followed by 2 weeks off	A: 23	A: 6 (26.1%)	A: 4 (17.4%)		A: 2 (8.7%)	

(7)	Domagala-Haduch et al. [24]	2016	Advanced renal cell carcinoma A: sunitinib: 50 mg/day for 4 weeks, and then it is stopped for 2 weeks	A: 39	A: 13 (33.3%)	A: 11 (28.2%)		A: 2 (5.1%)	

(8)	Goodman et al. [25]	2007	Refractory or intolerant gastrointestinal stromal tumors and advanced renal cell carcinoma A: sunitinib: 50 mg given daily for 4 weeks followed by a 2-week rest period (schedule 4/2)	A: 202	A: 58 (29%)	A: 56 (27.7%)		A: 2 (1%)	

(9)	Grünwald et al. [26]	2011	Metastatic renal cell carcinoma (RCC) IL-21 administered subcutaneously (s.c.) in combination with sunitinib 50 mg once daily (OD) orally at the 4 weeks on/2 weeks off A: rIL-21 3 *μ*g/kg B: rIL-21 10 *μ*g/kg	A: 5 B: 4	TOT: 4 (80%) TOT: 1 (25%)	A: 4 (80%) B: 1 (25%)		A: 0 B: 0	

(10)	Hong et al. [27]	2009	Advanced renal cell carcinoma patients A: sunitinib (50 mg for 4 weeks on/2 weeks off schedule) B: sunitinib 37.5 mg daily continuous dosing	A: 62 B: 14	TOT: 48 (63.2%)	TOT: 40 (64.5%)		TOT: 8 (10.5%)	

(11)	O'Donnell [28]	2011	Advanced renal cell carcinoma (RCC) A: sunitinib Seven patients started at a dose of 50 mg daily and nine patients started at a dose of 37.5 mg daily. The remaining three patients started at 25 mg daily	TOT: 19	A: 8 (42.1%)	A: 7 (36.9%)		A: 1 (5.26%)	

(12)	Kim et al. [29]	2014	The starting sunitinib dose was 37.5 and 50 mg for 12 and 22 patients, respectively. A 4 weeks on/2 weeks off regimen was followed for 31 patients; a 2 weeks on/2 weeks off regimen for one patient; and a daily regimen for two patients	A: 34	A: 13 (39%)	A: 10 (30%)		A: 3 (9%)	

(13)	Lee et al. [30]	2010	Metastatic renal cell carcinoma A: 6-week cycles of sunitinib treatment (50 mg once daily for 4 weeks on and 2 weeks off schedule)	A: 21	A: 9 (42.8%)	A: 5 (23.8%)		A: 4 (19%)	

(14)	Lee et al. [31]	2015	treatment-naïve patients with clear cell type metastatic renal cell carcinoma (mRCC) A: sunitinib 50 mg, “2 weeks on, 1 week off” B: sunitinib 50 mg, 4 weeks on, 2 weeks off	A: 38 B: 36	A: 27 (71%) B: 31 (86%)	A: 26 (68.4%) B: 27 (75%)		A: 1 (3%) B: 4 (11%)	

(15)	Lee et al. [32]	2013	A: SU: 37.5 mg/die + oral capecitabine 800 mg/m^2^ + cisplatin 60 mg/m^2^ B: SU: 37.5 mg/die + oral capecitabine 1,000 mg/m^2^ + cisplatin 60 mg/m^2^ C: SU: 25 mg/die + oral capecitabine 1,000 mg/m^2^ + cisplatin 80 mg/m^2^ D: SU: 37.5 mg/die + oral capecitabine 800 mg/m^2^ + oxaliplatin 110 mg/m^2^ E: SU: 37.5 mg/die + oral capecitabine 1,000 mg/m^2^ + oxaliplatin 110 mg/m^2^ F: SU: 25 mg/die + oral capecitabine 1,000 mg/m^2^ + oxaliplatin 110 mg/m^2^	A: 6 B: 7 C: 15 D: 23 E: 3 F: 22	A: 5 (83.3%) B: 3 (42.9%) C: 8 (53.3%) D: 10 (43.5%) E: 1 (33.3%) F: 7 (31.8%)	A: 5 (83.3%) B: 3 (42.9%) C: 6 (40%) D: 10 (43.5%) E: 1 (33.3%) F: 7 (31.8%)		A: 0 B: 0 C: 2 (13.3%) D: 0 E: 0 F: 0	

(16)	Lee et al. [6]	2009	Retrospective study A: sorafenib 400 mg twice daily for RCC and HCC B: sunitinib 50 mg daily, consisting of 4 weeks of treatment followed by a 2-week rest period in cycles of 6 weeks for RCC and GIST	A: 109 B: 119	A: 28 (26%) B: 43 (36%)	Not reported		Not reported	

(17)	Marschner et al. [33]	2017	mRCC A: sorafenib B: sunitinib	A: 25 B: 152	A: 3 (12.0%) B: 29 (23.2%)	A: 2 (8%) B: 27 (17.7%)		A: 1 (4.0%) B: 2 (1.6%)	

(18)	Mir et al. [34]	2016	mRCC A: sunitinib 50 mg once daily for 4 weeks	A: 50	A: 24 (12%)	A: 20 (40%)		A: 4 (2%)	

(19)	Patel et al. [35]	2009	Advanced renal cell carcinoma A: temsirolimus 15 mg was administered by intravenous (I.V.) infusion once weekly, and sunitinib 25 mg was administered orally once daily for 4 weeks, followed by a 2-week rest period.	A: 3	A: 1	A: 1 (33.3%)		A: 0	

(20)	Porta et al. [36]	2011	mRCC A: sunitinib B: sorafenib	A: 85 B: 60	A: 50 (58.8%) B: 16 (26.7%)	A: 48 (56.4%) B: 16 (26.7%)		A: 2 (2.4%) B: 0	

(21)	Rock et al. [37]	2007	GIST A: sunitinib 50 mg	A: 202	A: 58 (29%)	A: 56 (27.7%)		A: 2 (1%)	

(22)	Socinski et al. [38]	2008	Advanced non-small cell lung cancer A: sunitinib 50 mg/d for 4 weeks followed by 2 weeks of no treatment in a 6-week cycle	A: 63	A: 27 (43%)	A: 27 (43%)		A: 0	

(23)	Sonpavde et al. [39]	2010	Metastatic castration-resistant prostate cancer A: sunitinib 50 mg/day: 4 weeks on followed by 2 weeks off	A: 36	A: 2 (5.7%)	A: 1		A: 1	

(24)	Sternberg et al. [40]	2015	Metastatic renal cell carcinoma (mRCC) A: oral sunitinib 50 mg/day on a 4 weeks on/2 weeks off schedule	A: 521	A: 192 (37%)	A: 159 (31%)		A: 33 (6%)	

(25)	Van Der Veldt et al. [41]	2008	Advanced RCC A: sunitinib 50 mg daily for 4-week treatment followed by 2-week rest period in a cycle of 6 weeks	A: 82	A: 58 (70.73%)	A: 51 (62%)		A: 7 (9%)	

(26)	Yildiz et al. [42]	2011	Advanced renal cell carcinoma A: sunitinib 37.5 mg daily B: sunitinib 25 mg	A: 67	A: 36 (51%)	A: 36 (51%)		A: 0	

(27)	Yoo et al. [43]	2010	Renal cell carcinoma A: sunitinib 50 and 37.5 mg daily	A: 65	A: 37 (57%)	A: 31 (50%)		A: 6 (10%)	

(28)	Zhao et al. [44]	2013	Locally advanced clear cell renal carcinoma A: sorafenib 400 mg orally twice daily for a 4-week cycle B: sunitinib 50 mg orally daily for a 6-week cycle	A: 20 B: 23	A: 8 (40%) B: 7 (30%)	Not reported		Not reported	

	Total			2.596	914 (35.2%)				

	Total with grade			2.068	829 (40.08%)	739 (35.73%)		90 (5.35%)	

	Total not reporting grade^*∗*^			142	50 (3.52%)	Not reported		Not reported	

	Total reporting only grade >2^*∗∗*^			386	35 (0.06%)	Not reported		35 (0.06%)	

*Note*. ^*∗*^Carrato et al. (2013) [22] did not report the grade of stomatitis; ^*∗∗*^Lee et al. (2009) [6] and Zhao et al. (2013) [44] reported the incidence rates limited to grade 3 and grade 4 treatment-related toxicities; for this reason, data about cases of stomatitis and stomatitis grades 1 and 2 are lower than real.

**Table 2:** Report on all papers about sorafenib and stomatitis.

	Authors	Year	Neoplasia	Cases number	Stomatitis (%)	Stomatitis grade 1%	Stomatitis grade 2%	Stomatitis grade 3%	Stomatitis grade 4%
(1)	Cho et al. [45]	2013	Advanced hepatocellular carcinoma A: sorafenib 400 mg twice daily	A: 99	A: 4 (4%)	Not reported		Not reported	

(2)	Chrisoulidou et al. [46]	2015	Refractory thyroid cancer Sorafenib 400 mg was given orally twice daily continuously, sunitinib 50 mg was given once daily on a 4 weeks of treatment followed by 2-week intervals without therapy, and vandetanib 300 mg was given once daily	A: 24	A: 13 (54%)	A: 12 (50%)		A: 1 (4.16%)	

(3)	Grignani et al. [47]	2015	Unresectable high-grade osteosarcoma progressing after standard treatment 400 mg sorafenib twice a day together with 5 mg everolimus once a day	A: 38	A: 20 (52.63%)	Not reported		Not reported	

(4)	Hainsworth et al. [48]	2015	Stage III/IV epithelial ovarian cancer A: paclitaxel 175 mg/m2, 1–3 h IV infusion/carboplatin AUC 6.0, 20 min IV infusion/sorafenib 400 mg PO BID	A: 43	A: 16 (37%)	Oral mucositis A: 16 (37%)		A: 0	

(5)	Hainsworth et al. [49]	2013	Phase II Sorafenib 200 mg PO BID and everolimus 35 mg PO once weekly	A: 75	A: 10 (13.3%)	Mucositis/stomatitis: 10/14% Mucositis/stomatitis: 2/3%		Mucositis/stomatitis: 0 Mucositis/stomatitis: 0	

(6)	Lee et al. [6]	2009	Retrospective study A: sorafenib 400 mg twice daily for RCC and HCC B: sunitinib 50 mg daily, consisting of 4 weeks of treatment followed by a 2-week rest period in cycles of 6 weeks for RCC and GIST	A: 109 B: 119	A: 28 (26%) B: 43 (36%)	Not reported		Not reported	

(7)	Marschner et al. [33]	2017	mRCC A: sorafenib B: sunitinib	A: 25 B: 152	A: 3 (12.0%) B: 29 (23.2%)	A: 2 (8%) B: 27 (17.7%)		A: 1 (4.0%) B: 2 (1.6%)	

(8)	Meyer et al. [50]	2017	Unresectable HCC A: sorafenib 660 mg	A: 157	A: 41 (26%)	A: 36 (23%)		A: 5 (3%)	

(9)	Porta et al. [51]	2011	mRCC A: sunitinib B: sorafenib	A: 85 B: 60	A: 50 (58.8%) B: 16 (26.7%)	A: 48 (56.4%) B: 16 (26.7%)		A: 2 (2.4%) B: 0	

(10)	Richly et al. [52]	2006	Refractory solid tumors A: sorafenib 100 mg + doxorubicin B: sorafenib 200 mg + doxorubicin C: sorafenib 400 mg + doxorubicin D: sorafenib 400 mg + doxorubicin	A: 6 B: 6 C: 12 D: 10	TOT 11 (32%)	Not reported		A: 3 (50%) B: - C: 6 (50%) D: 2 (20%)	

(11)	Schwartzberg et al. [53]	2013	Advanced breast cancer A: sorafenib (400 mg, twice daily)	A: 79	A: 27 (34.1%)	A: 17 (21.5%)		A: 10 (12.65%)	

(12)	Shacham-Shmueli et al. [54]	2012	Advanced solid tumors A: sorafenib 100 mg BID (50 mg tablets) + infusion regimen B: sorafenib 200 mg BID (50 mg tablets) + infusion regimen C: sorafenib 400 mg BID (50 mg tablets) + infusion regimen D: sorafenib 400 mg BID (50 mg tablets) + bolus A regimen E: sorafenib 400 mg BID (200 mg tablets) + infusion regimen F: sorafenib 400 mg BID (200 mg tablets) + bolus B regimen	A: 10 B: 7 C: 6 D: 9 E: 6 F: 9	A: - B: - C: - D: 3 (33%) E: - F: -	Not reported		A: - B: - C: - D: 3 (33%) E: - F: -	

(13)	Sho et al. [55]	2017	Advanced hepatocellular carcinoma A: 250 mg/m^2^ of 5-FU and sorafenib 800 mg daily B: 350 mg/m^2^ of 5-FU and sorafenib 800 mg daily C: 450 mg/m^2^ of 5-FU and sorafenib 800 mg daily	A: 3 B: 3 C: 6	A: 0 B: 1 C: 3	A: 0 B: 1 C: 2		A: - B: - C: 1	

(14)	Ueda et al. [56]	2013	Metastatic renal cell carcinoma A: axitinib B: sorafenib	A: 359 B: 355	A: 54 (15.04%) B: 44 (12.39%)	A: 49 (13.64%) B: 43 (12.11%)		A: 5 (1.39%) B: 1 (0.28%)	

(15)	Williamson et al. [57]	2010	Advanced and metastatic squamous cell carcinoma of the head and neck A: sorafenib orally at 400 mg twice daily on continuous basis in 28-day cycles	A: 41	A: 2 (4.9%)	Not reported		A: 2	

(16)	Zhao et al. [44]	2013	Locally advanced clear cell renal carcinoma A: sorafenib 400 mg orally twice daily for 4-week cycle B: sunitinib 50 mg orally daily for a 6-week cycle	A: 20 B: 23	A: 8 (40%) B: 7 (30%)	Not reported		Not reported	

	Total			1218	250 (20.52%)				

	Total with grade			830	174 (20.96%)	155 (18.67%)		19 (2,28%)	

	Total not reporting grade^*∗*^			266	60 (22.55%)	Not reported		Not reported	

	Total reporting only grade >2^*∗∗*^			122	16 (13.11%)	Not reported		16 (13.11%)	

*Note*. ^*∗*^Cho et al. (2013) [45], Grignani et al. (2015) [47], Lee et al. (2009) [6], and Zhao et al. (2013) [44] did not report the grade of stomatitis; ^*∗∗*^Richly et al. (2006) [52], Shacham-Shmueli et al. (2012) [54], and Williamson et al. (2010) [57] reported the incidence rates limited to grade 3 and grade 4 treatment-related toxicities; for this reason, data about cases of stomatitis and stomatitis grades 1 and 2 are lower than real.

**Table 3:** Report on all papers about axitinib and stomatitis.

	Authors	Year	Neoplasia	Number of cases	Stomatitis number	Stomatitis grade 1	Stomatitis grade 2	Stomatitis grade 3	Stomatitis grade 4
(1)	Rugo et al. [58]	2005	Phase I study Advanced solid tumors A: AG-013736 Axitinib 5–30 mg in 28-day cycle	A: 36	A: 4 (11%)	A: 2 (6%)		A: 2 (6%)	

(2)	Ueda et al. [56]	2013	Metastatic renal cell carcinoma A: axitinib B: sorafenib	A: 359 B: 355	A: 54 (15.04%) B: 44 (12.39%)	A: 49 (13.64%) B: 43 (12.11%)		A: 5 (1.39%) B: 1 (0.28%)	

(3)	Karam et al. [59]	2014	Phase II trial of locally advanced nonmetastatic clear cell renal carcinoma A: axitinib 5 mg for up to 12 weeks	A: 24	A: 17 (70.8%)	A: 16 (67%)		A: 1 (4.2%)	

(4)	Oh et al. [60]	2015	Phase I study: previously untreated advanced gastric cancer A: axitinib 5 mg twice a day (days 1 to 21) with cisplatin 80 mg/m^2^ (day 1) and capecitabine 1,000 mg/m^2^ twice a day (days 1 to 14) in 21-day cycles	A: 22	A: 16 (72.7%)	A: 12 (54.5%)		A: 4 (18.2%)	

	*Total*			*441*	*91 (20.63%)*	*79 (17.91%)*		*12 (2.72%)*	

**Table 4:** Report on all papers about cabozantinib and stomatitis.

	Authors	Year	Neoplasia	nNumber of cases	Stomatitis total	Stomatitis grade 1	Stomatitis grade 2	Stomatitis grade 3	Stomatitis grade 4
1	Neal et al. [61]	2016	Phase II trial for EGFR wild-type non-small-cell lung cancer A: erlotinib B: cabozantinib C: erlotinib + cabozantinib	A: 40 B: 40 C: 39	A: 2 (5%) B: 17 (43%) C: 9 (24%)	A: 2 (5%) B: 13 (33%) C: 8 (21%)		A: 0 B: 4 (10%) C: 1 (3%)	A: 0 B: 0 C: 0

2	Tolaney et al. [62]	2017	Phase II metastatic triple negative breast cancer A: oral dosing of cabozantinib at 60 mg daily over a 21-day cycle	A: 35	A: 13 (37%)	A: 11	A: 2	A: 0	A: 0

	*Total*			*114*	*39 (34.21%)*	*34 (29.82%)*		*5 (4.38%)*	
